# Effect of temperature and AC field duration on the dielectric behavior of PVA/GO/FeGaInS_4_ nanocomposites

**DOI:** 10.1039/d5ra06744h

**Published:** 2025-12-10

**Authors:** Zeynab Addayeva, Mustafa Muradov, Goncha Eyvazova, Namiq Niftiyev, Faik Mammadov

**Affiliations:** a Baku State University, Nano Research Laboratory Baku Azerbaijan; b Baku State University, Faculty of Physics, Department of Physics of Semiconductor Azerbaijan; c Azerbaijan State Pedagogical University Baku Azerbaijan; d Institute of Catalysis and Inorganic Chemistry Baku Azerbaijan

## Abstract

This study explores the dielectric behavior of polyvinyl alcohol (PVA)-based nanocomposites incorporating 2 wt% FeGaInS_4_ and 3 wt% graphene oxide (GO), focusing on the effects of temperature and alternating current (AC) electric field exposure duration. The nanocomposites were synthesized *via* ultrasonic dispersion in water, followed by casting and ambient drying. X-ray diffraction (XRD) confirmed the preservation of the FeGaInS_4_ crystalline phase and the disordered, exfoliated state of GO within the polymer matrix. Dielectric spectroscopy, performed across 120 Hz to 1 MHz and temperatures between 40 and 80 °C, revealed a decrease in dielectric constant (*ε*′) with frequency, attributed to interfacial and dipolar polarization mechanisms. With increasing temperature, *ε*′ rose due to enhanced chain mobility and interfacial polarization. Notably, 2 h of AC field exposure at 40 °C improved both *ε*′ and dielectric loss (tan *δ*), while prolonged exposure led to relaxation effects and reduced performance. Activation energy (*E*_a_), calculated using the correlated barrier hopping (CBH) model, decreased from 0.75 to 0.40 eV at 500 Hz with longer field exposure, indicating improved charge hopping. At higher frequencies (50 kHz), *E*_a_ showed a transient increase before stabilization. The results demonstrate the tunability of dielectric properties *via* AC field treatment, highlighting the potential of these nanocomposites for applications in flexible electronics and dielectric energy storage.

## Abstract 2

This study explores the dielectric behavior of polyvinyl alcohol (PVA)-based nanocomposites incorporating 2 wt% FeGaInS_4_ and 3 wt% graphene oxide (GO), focusing on the effects of temperature and AC electric field exposure duration.

## Graphical abstract

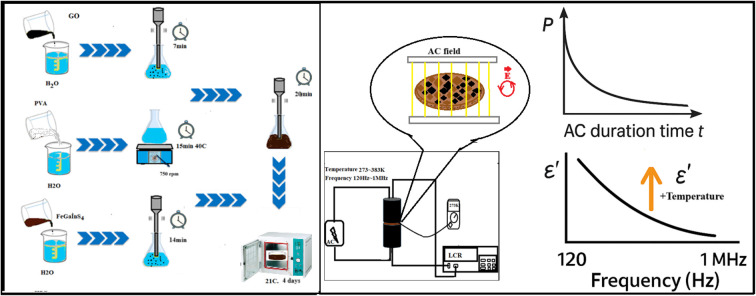

## 1 Introduction

Dielectric polymers and their modern derivatives, namely polymer composites, are functional materials of particular importance due to their wide applications in electronics and optoelectronics. Their high electrical insulation, mechanical flexibility, low density, cost-effective manufacturing, and ease of processing enable their extensive utilization in sensors, capacitors, energy storage devices, flexible electronics, and smart material systems.^1–6^ In practical conditions, polymer-based insulators are subjected to multiple external stimuli, such as electric and magnetic fields, temperature fluctuations, UV and gamma radiation, mechanical stress, and pressure. While short-term exposure may not significantly alter their physicochemical properties, long-term or repeated stresses can induce structural deformations, increased electrical conductivity, and degradation in dielectric performance.^7–14^

One of the key mechanisms in polymers under alternating current (AC) fields is dipolar polarization, where polar groups (*e.g.*, –OH, –C=O, –Cl) align with the electric field, generating internal friction, heat, increased dielectric losses, and eventual electrical fatigue.^15–19^ In composites, Maxwell–Wagner–Sillars (MWS) polarization plays a significant role, leading to charge accumulation at interfaces and local stress concentrations, which can promote microcracks and directional conductive pathways, especially at low frequencies.^20–25^ Space charge accumulation, driven by ion and defect migration under electric fields, further contributes to localized heating and conductive pathways formation, while prolonged electrical and thermal stress enhances polymer chain mobility, chain scission, and creep, reducing mechanical strength and insulation stability.^26–29^

In this study, PVA was selected as the matrix due to its water solubility, flexibility, biocompatibility, and sensitivity to interfacial modification, allowing tunable dielectric behavior upon filler addition. Two layered materials were incorporated to enhance interfacial polarization: FeGaInS_4_ crystals as a micro-filler and GO as a nano-filler, with GO penetrating interlayer spaces to further promote polarization.^30–32^ The distinct band gaps of these fillers influence charge carrier mobility differently, affecting the response to applied electric fields.

FeGaInS_4_ layered crystals were synthesized *via* the Bridgman technique. This quaternary metal chalcogenide crystallizes in the rhombohedral system (R3m, No. 160) with refined lattice parameters *a* = *b* = 5.406 Å and *c* = 10.708 Å, exhibiting a highly symmetric and anisotropic layered structure. Fe, Ga, and In atoms occupy distinct coordination sites, forming tetrahedral bonds with sulfur, creating a complex three-dimensional network that significantly affects electrical conductivity, optical absorption, dielectric behavior, and thermal stability.^33–36^ Its anisotropic layered architecture allows facile exfoliation into few-layer or monolayer nanosheets, highlighting potential for 2D material applications and high-performance composites.^24,25^

Graphene oxide is renowned for its high surface area, abundance of functional groups, and excellent mechanical properties. GO incorporation into PVA enhances interfacial charge interactions, promotes polymer–filler interactions, and forms secondary interphase regions. The combined use of FeGaInS_4_ and GO is expected to yield synergistic effects, improving dielectric stability, reducing dielectric loss, and maintaining functional stability under thermal stress.^37–40^

This work systematically investigates the dielectric properties of PVA/GO/FeGaInS_4_ nanocomposites with varying filler concentrations under alternating electric fields across different temperatures and durations. The results advance fundamental understanding and provide insights for designing stable nanocomposites for energy storage, sensors, and flexible electronics. Future studies may explore higher filler loadings, alternative polymer matrices, and diverse electrical stress conditions to further optimize performance.

## 2 Materials and methods

### 2.1 Synthesis of FeGaInS_4_/PVA/GO composites

To prepare the FeGaInS_4_/PVA/GO composites, FeGaInS_4_ crystals and graphene oxide (GO) were first synthesized independently, then incorporated into a 5 wt% polyvinyl alcohol (PVA) solution using a casting solution technique.

GO was produced using a modified version of Hummer's method. In this process, 3 g of graphite powder and 1.5 g of sodium nitrate were mixed in a 500 mL beaker. While maintaining the mixture in an ice bath, 70 mL of concentrated sulfuric acid (H_2_SO_4_) was slowly added, and the resulting solution was stirred for 1 hour. Potassium permanganate (KMnO_4_) was then introduced gradually, keeping the reaction temperature below 20 °C. After stirring for 3 hours under controlled conditions, the mixture was further stirred at 35 °C for an additional hour. Next, 150 mL of water was added dropwise, causing the viscosity to increase and the temperature to rise to 98 °C, where it was held for 30 minutes. Following this, the mixture was diluted with 300 mL of water, stirred for 1 hour, and treated with 15 mL of 30 wt% hydrogen peroxide (H_2_O_2_) for 30 minutes. The resultant material was filtered and washed with a 1 : 10 hydrochloric acid (HCl) to distilled water solution (250 mL) to eliminate remaining metal ions. The filtered product was air-dried at room temperature, redispersed in distilled water, sonicated, centrifuged, filtered again, and finally dried under ambient conditions.^41–43^

For the polymer matrix, a 5 wt% PVA aqueous solution was prepared. FeGaInS_4_ crystal powder was added to water and dispersed for 14 minutes. Separately, graphene oxide (GO) was dispersed in water for 7 minutes. These two dispersions were then combined with the 5 wt% PVA solution and further dispersed for 20 minutes to ensure uniform mixing. The final composite contained 2 wt% GO (relative to PVA) and 3 wt% FeGaInS_4_.The final mixture was cast into Petri dishes and dried at room temperature (21 °C) under a vacuum of 0.2 MPa; the drying process lasted for four days. After drying, the samples were placed into the sample holder of the E7-20 dielectric spectrometer. Initially, dielectric measurements were conducted at room temperature. Subsequently, the temperature was raised to 40 °C, and measurements were performed at one-hour intervals. This procedure was repeated at 60 °C and 80 °C to systematically investigate the influence of both time and temperature on the dielectric properties of the polymer composite.

### 2.2 Characterization of FeGaInS_4_/PVA/GO composites

The structure and dielectric behavior of the resulting composite films were studied using X-ray diffraction (XRD) and dielectric spectroscopy. XRD measurements were carried out using a Rigaku MiniFlex 600 diffractometer with Ni-filtered Cu-Kα radiation (*λ* = 1.5406 Å). Dielectric properties were examined using an E7-20 dielectric spectrometer, operating across a frequency range of 1.2 × 10^2^ to 10^6^ Hz and a temperature span from 293 K to 373 K. These characterization techniques collectively provided detailed insight into the structural and dielectric performance of the FeGaInS_4_/PVA/GO composites. FeGaInS_4_ microcrystals and graphene oxide (GO) were dispersed in an aqueous PVA solution using a Qsonica Q700 ultrasonic processor (700 W, 20 kHz).

## 3 Results and discussion

### 3.1 XRD analysis

**Fig. 1:**
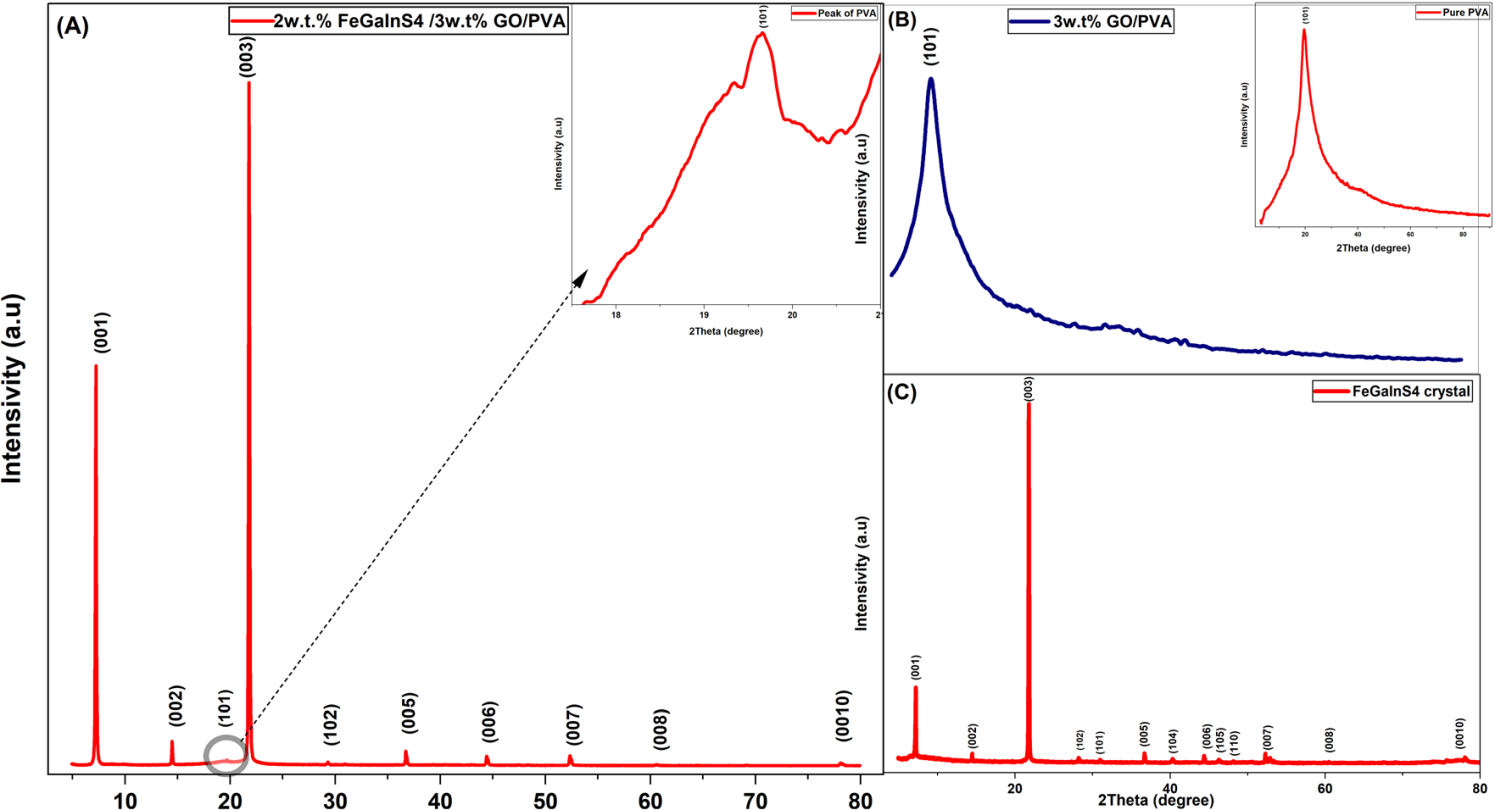
X-ray diffraction pattern of (A) 2wt%FeGaInS_4_/3wt% GO/PVA, (B) PVA/GO, (C) pure PVA and (D) FeGaInS_4_ crystal.

X-ray diffraction (XRD) analysis was performed to investigate the crystalline structure of the FeGaInS_4_ crystal and the FeGaInS_4_/GO/PVA composites synthesized at room temperature. Fig. 1A shows the X-ray diffraction pattern of 2wt%FeGaInS_4_/3wt% GO/PVA, while Fig. 1B displays the diffraction pattern of the GO/PVA, Fig. 1C displays the diffraction pattern of pure PVA Fig. 1D displays the diffraction pattern of the FeGaInS_4_ crystals.

The XRD patterns of the synthesized composites exhibit distinct diffraction peaks at 2*θ* = 7.15°, 14.41°, 21.72°, 28.2°, 37°, 40.8°, 44.74°, 52.63°, 61.2°, and 78.6°. These reflections correspond to the (001), (002), (003), (102), (005), (006), (007), (008), and (0010) planes of FeGaInS_4_, which are in good agreement with previously reported data.^44,45^ The sharp and well-defined peaks confirm that FeGaInS_4_ maintains a high degree of crystallinity even after incorporation into the polymeric matrix. Furthermore, the absence of any significant peak shifts or intensity variations upon the addition of PVA and GO indicates that the FeGaInS_4_ phase remains structurally stable. This observation suggests that no phase transformation or lattice distortion occurs within the composite system.

In addition to the FeGaInS_4_-specific reflections, all composite samples exhibit a broad diffraction peak centered at approximately 2*θ* ≈ 19.3°, corresponding to the (101) plane of PVA. This feature, also evident in the pure PVA diffractogram (Fig. 1C), is characteristic of the semi-crystalline nature of PVA.^46^ The persistence of this peak across all samples confirms the successful incorporation of PVA into the hybrid structure.

A comparison between the GO/PVA (Fig. 1B) and pure PVA (Fig. 1C) diffractograms reveals that no distinct peak associated with graphene oxide is present. The absence of the characteristic GO (001) reflection near 2*θ* ≈ 10°^43,47,48^ can be attributed to the high degree of exfoliation and structural disorder of GO within the PVA matrix. Moreover, the strong encapsulation of GO sheets by the PVA chains effectively covers their surface, making the detection of the GO peak more difficult. Such exfoliation results from strong interfacial interactions between GO nanosheets and PVA macromolecules, leading to the disappearance of the ordered layered structure of GO. This observation supports the formation of a well-dispersed and molecularly homogeneous GO/PVA hybrid network, which facilitates the uniform incorporation of FeGaInS_4_ nanocrystals within the polymer matrix. The absence of the GO (001) peak in the XRD patterns, while the characteristic D and G bands of GO are clearly observed in the Raman spectra, supports the presence of well-dispersed and exfoliated GO within the PVA matrix.^49,50^ However, it should be noted that complete XRD peak disappearance can also result from low GO content, strong structural disorder, or overlap with the polymer background.

### 3.2 Dielectric spectroscopy

**Fig. 2:**
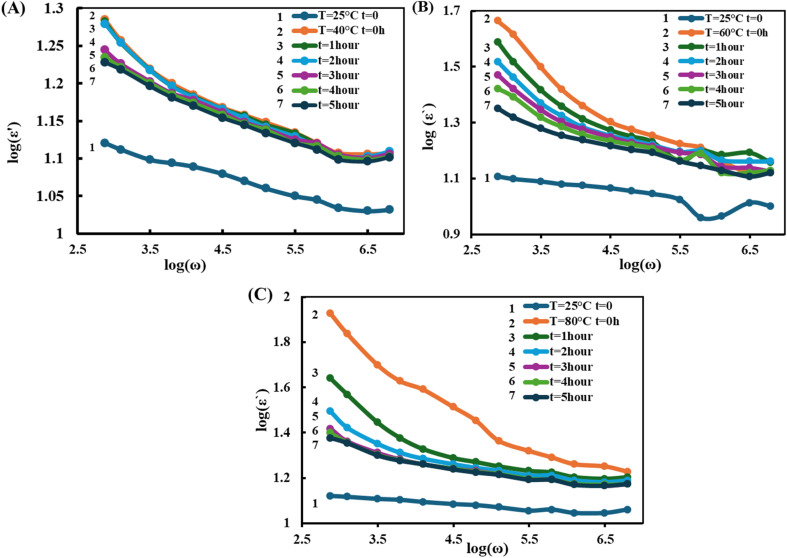
Frequency dependence of the dielectric permittivity (*ε*′) of the PVA + 3wt% GO + 2wt% FeGaInS_4_ composite under different durations of AC electric field exposure (0–5 hours) at various temperatures: (A) 40 °C, (B) 60 °C, and (C) 80 °C.

Dielectric measurements were conducted under an alternating electric field within the frequency range of 120 Hz to 1 MHz. Fig. 2 presents the frequency dependence of the dielectric permittivity (*ε*′) for the PVA/3wt%GO/2wt%FeGaInS_4_ composite at different temperatures (40 °C, 60 °C, and 80 °C) and exposure durations to the AC electric field (0–5 hours).

In all the depicted graphs (40 °C, 60 °C, and 80 °C), the dielectric constant (*ε*′) decreases with increasing frequency. At low frequencies, Maxwell–Wagner interfacial polarization plays a dominant role; however, at higher frequencies, this mechanism is suppressed by dipolar polarization. As the electric field oscillates more rapidly, molecular dipoles are unable to reorient in time, resulting in a decline in *ε*′.^51,52^

In all three temperature measurements, a clear increasing trend in dielectric permittivity (*ε*′) is observed with rising temperature. At 40 °C, the initial value of *ε*′ lies within the range of 1.12–1.15 and gradually decreases to approximately 1.00–1.05 as the frequency increases from 10^2^ Hz to 10^6^ Hz (Fig. 2A). At 60 °C, the initial *ε*′ ranges between 1.30 and 1.50, decreasing to around 1.10–1.20 with increasing frequency (Fig. 2B). At 80 °C, the initial *ε*′ reaches as high as 1.40–1.90, indicating a significant enhancement in dielectric permittivity due to strong interfacial polarization facilitated by the elevated temperature (Fig. 2C).

This increase in *ε*′ with temperature is attributed to enhanced molecular mobility and intensified interfacial polarization. Moreover, elevated temperature promotes the plasticization of polymer chains, facilitating the penetration of PVA molecules into the interlayer spaces of FeGaInS_4_ crystals. This expansion of the interfacial area strengthens interphase polarization.

As shown in Fig. 2A, an increase in temperature from 25 °C to 40 °C results in an increase in dielectric permittivity, which can be explained by temperature-induced enhancement in polymer–filler interactions.^24,25^ However, at the constant temperature of 40 °C, *ε*′ tends to decrease over time. One hour after the initial measurement, *ε*′ drops below its initial value. This behavior is associated with the use of water as a solvent during composite preparation and subsequent drying at room temperature, which may leave residual water molecules within the mesopores of the polymer matrix. At low temperatures, water molecules near the surface escape from the environment. Since water is a polar molecule, its loss leads to a decrease in the overall polarizability of the medium.

Interestingly, after 2 hours of exposure to an electric field at 40 °C, *ε*′ sharply increases—especially at low frequencies—reaching a maximum value. This behavior can be attributed to temperature-induced weakening of certain polymer bonds, which enhances the interaction between the polymer and the filler. As a result, the interphase structure is re-formed with fewer defects, thereby increasing interphase polarization.

With further time progression, polarization begins to decline. This is likely due to the gradual fatigue of dipoles and delayed relaxation processes under prolonged exposure to the external electric field. Long-term application of an AC electric field can lead to the formation of micro-defects and trapping sites around the filler and at interphases. These trap states facilitate space charge accumulation, which generates internal electric fields. These internal fields act against the external field, weakening its influence and thereby diminishing polarization through a relaxation suppression mechanism.

Since reaching 60 °C and 80 °C from room temperature takes a certain amount of time, it can be inferred that during this period, the interphase structure has already been reformed. The graph clearly shows that dielectric permittivity decreases progressively over time. As the temperature increases-particularly approaching the glass transition temperature—the polymer chains become more flexible due to the weakening of intermolecular interactions. This facilitates improved dispersion of the filler, enhances its distribution within the matrix, and allows polymer chains to more effectively penetrate the interlayer spaces of the filler. Consequently, the interfacial area increases.

An enlarged interfacial region implies a greater number of charge-trapping sites—also known as energetic traps. Therefore, at elevated temperatures, the onset of dielectric fatigue within the polymer occurs sooner. The increased temperature activates these energetic traps, generating internal electric fields. These internal fields oppose the external electric field, thereby reducing the alignment efficiency of dipoles along the field direction. As a result, polarization is suppressed.

**Fig. 3:**
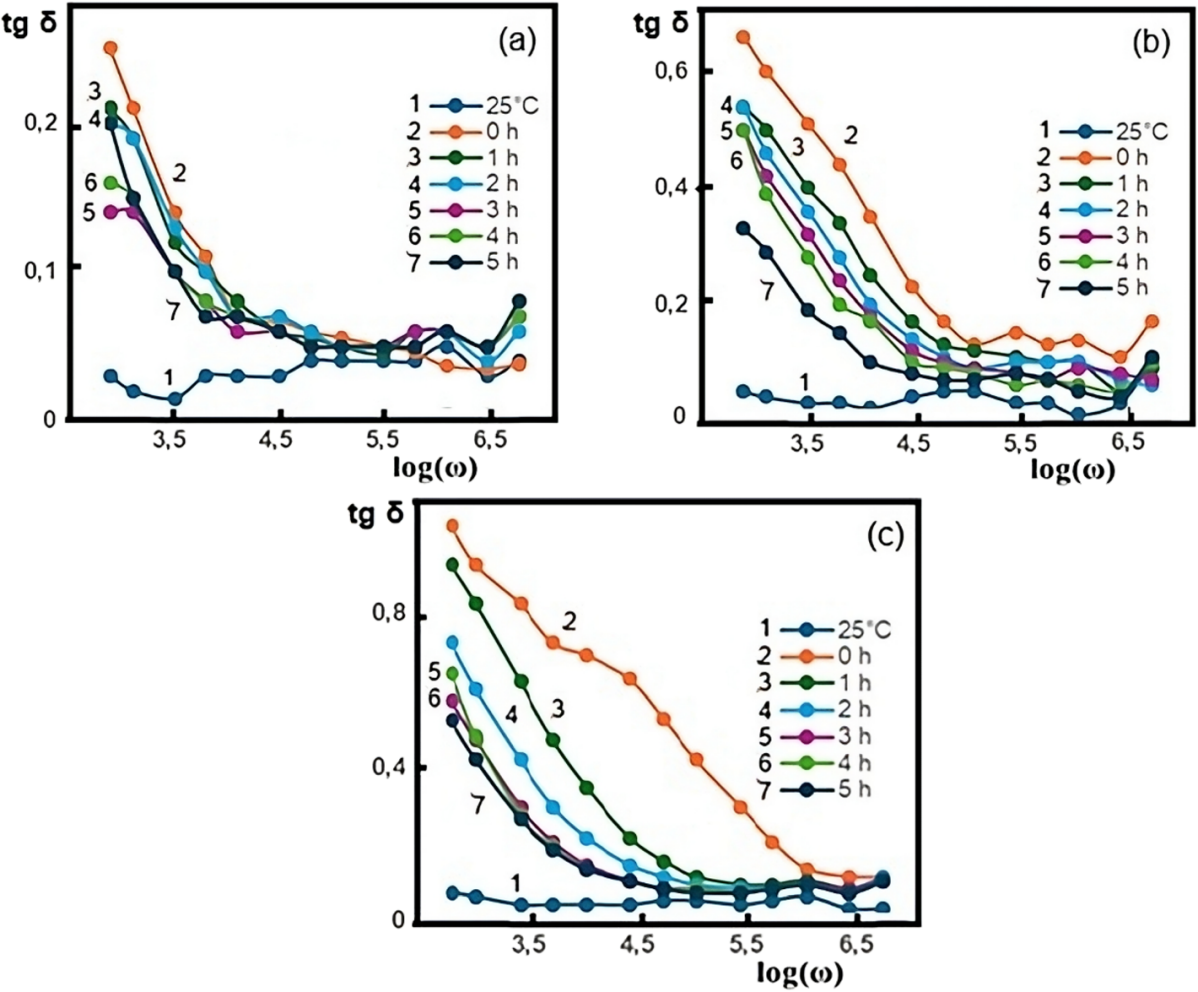
Frequency dependence of the dielectric loss tangent (tan *δ*) of the PVA + 3wt% GO + 2wt% FeGaInS_4_ composite under different durations of AC electric field exposure (0–5 hours) and at various temperatures: (a) 40 °C, (b) 60 °C, (c) 80 °C.

Fig. 3 shows the frequency dependence of the dielectric loss tangent (tg *δ*) for the PVA/3wt% GO/2wt% FeGaInS_4_ composite at different temperatures and under varying durations of AC electric field exposure.

At all three temperatures (40 °C, 60 °C, and 80 °C), the loss tangent (tg *δ*) begins with relatively high values at low frequencies and gradually decreases as the frequency increases. This behavior is attributed to the delayed response of interfacial regions and dipolar relaxations.^53^ When compared with previous studies, it can be observed that no distinct tg *δ* maximum is present within the measured frequency range; instead, there is a tendency for the peak to shift toward lower frequencies.^25^ This shift is directly associated with the incorporation of GO into the polymer composite. Since the tg *δ* maximum is inversely proportional to the relaxation time, this shift indicates an increase in the relaxation time.

The minimum value of tg *δ* is recorded at room temperature. As the temperature increases to 40 °C, thermal motion of the dipoles becomes more intense, leading to higher thermal energy and, consequently, an increase in tg *δ*. Over time, a decrease in tg *δ* is observed within the polymer composite. This behavior is likely due to temperature- and time-induced redistribution of filler particles toward a more homogeneous state within the polymer matrix. Such redistribution restricts the ability of polymer chains to orient along the electric field direction, resulting in a reduction of energy loss associated with dipole alignment and, therefore, lower dielectric loss.

This time-dependent decrease in dielectric loss becomes more pronounced at higher temperatures. Moreover, as the temperature approaches the glass transition temperature of PVA, the curves become more regular. This is attributed to increased segmental mobility of polymer chains, allowing them to more easily align with the external electric field.

**Fig. 4:**
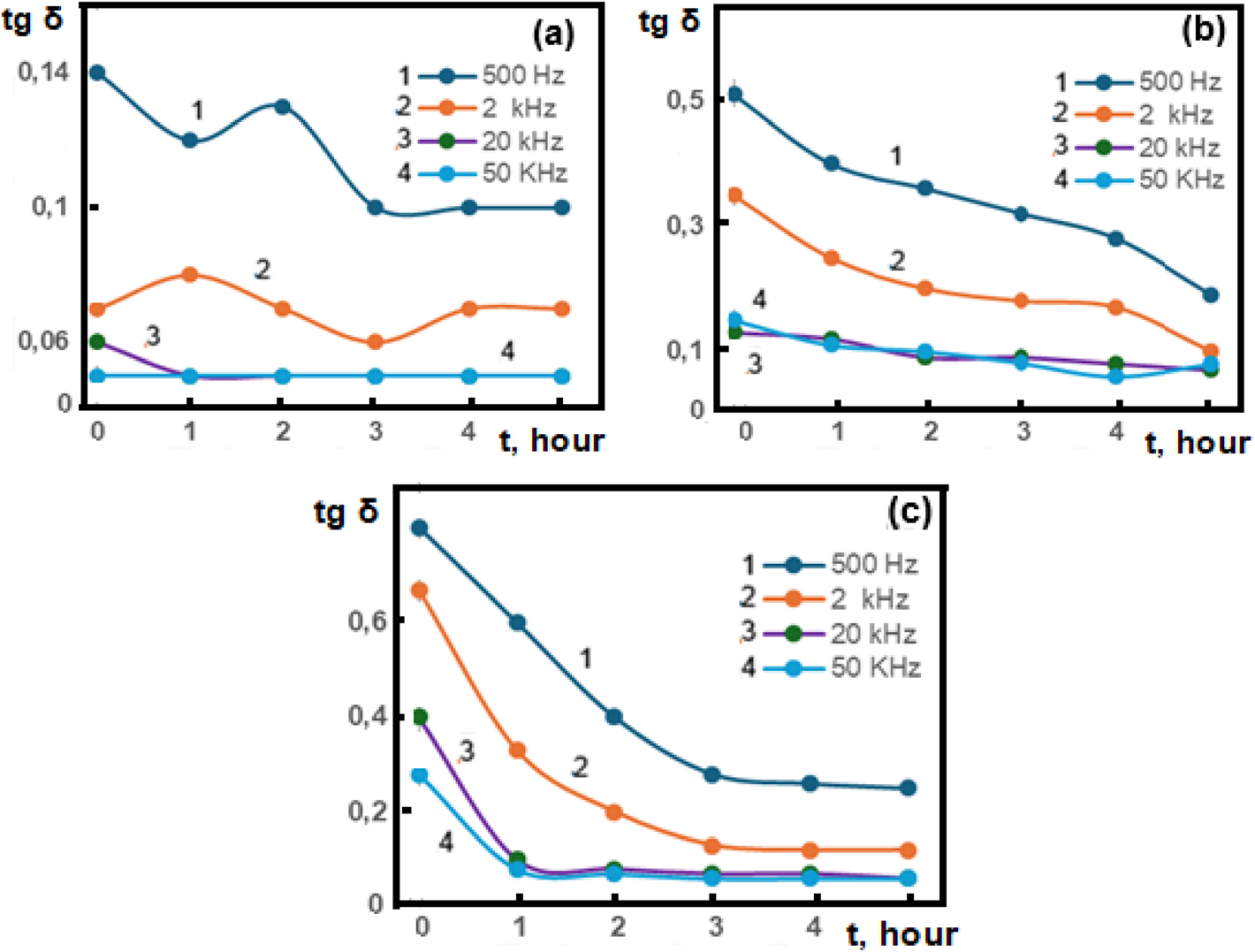
Dependence of the dielectric loss tangent (tg δ) of the PVA + 3wt% GO + 2wt% FeGaInS_4_ composite on the duration of AC electric field exposure (0–5 hours) at various temperatures and frequencies: (a) 40 °C, (b) 60 °C, (c) 80 °C.

Fig. 4 illustrates the dependence of the loss tangent (tg δ) on exposure time for the polymer composite measured at various frequencies (500 Hz–50 kHz) and temperatures of 40 °C, 60 °C, and 80 °C.

It is evident from the figure that at low frequencies, tg *δ* exhibits higher values and gradually decreases with increasing frequency. This behavior can be explained by the dominance of interfacial polarization (Maxwell–Wagner–Sillars) at low-frequency regions and the energy dissipation occurring during dipolar relaxation processes.

At 40 °C, the material exhibits a three-stage response under AC field exposure. During the first hour (0–1 h), thermal deaging and the applied field induce structural relaxation, leading to a decrease in the loss tangent (tg *δ*).^54^ In the second stage (1–2 h), interfacial restructuring enhances polymer–filler interactions, resulting in maximum polarization.^55–57^ From 2–5 h, dielectric fatigue and stabilization occur as repeated charge accumulation reduces tg *δ* to a minimum.^58,59^ At higher temperatures, such as 60 °C and 80 °C, accelerated kinetics dominate,^60,61^ allowing the system to overcome activation barriers more rapidly and bypass the anomaly observed at 40 °C, progressing directly toward equilibrium.

At these elevated temperatures, close to the polymer's glass transition, increased polymer fluidity initially leads to higher dielectric loss values; however, tg *δ* decreases over time. The temperature facilitates local charge accumulation around well-dispersed filler particles, generating internal fields that weaken the external electric field, which impedes dipole rotation and reduces energy dissipation. Additionally, agglomeration effects contribute to this decrease, as volumetrically larger filler clusters have restricted rotation under the field, resulting in lower heat generation during polarization and thus a reduced loss tangent.

**Fig. 5:**
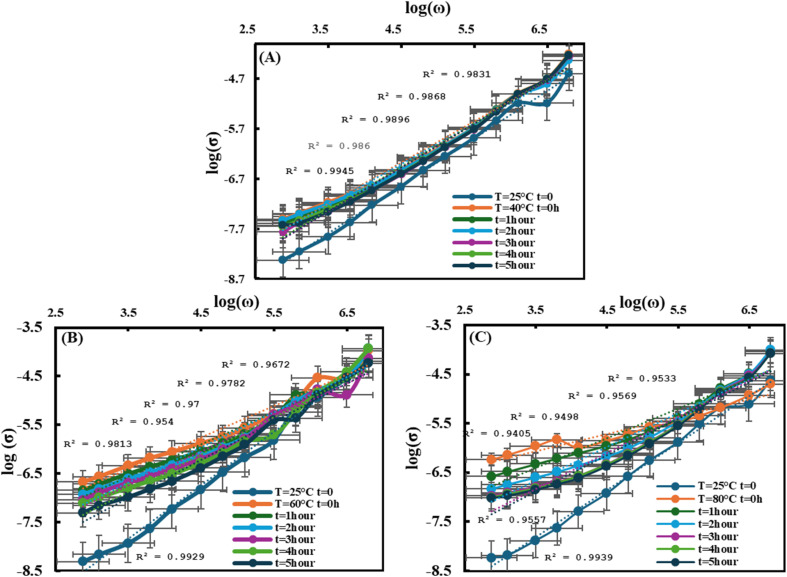
Frequency dependence of the electrical conductivity of the PVA + 3wt% GO + 2wt% FeGaInS_4_ composite at various temperatures and durations of AC electric field exposure (0–5 hours): (A) 40 °C, (B) 60 °C, (C) 80 °C.

Fig. 5 shows the frequency dependence of electricaln conductivity for PVA/3wt% GO/2wt% FeGaInS_4_ samples measured at various frequencies and temperatures of 40 °C, 60 °C, and 80 °C.

It is evident that at all three temperatures, the electrical conductivity increases with rising frequency. This behavior follows Jonscher's universal power law, *σ*_ac_(*ω*) = *Aω*^S^ and arises from the hopping of charge carriers between localized states, as described by the Correlated Barrier Hopping (CBH) mechanism.^62^ The non-linear behavior observed in the Arrhenius plots of the PVA/GO/FeGaInS_4_ nanocomposites can be attributed to the CBH mechanism and frequency-dependent charge carrier dynamics. In this framework, the hopping barrier and distance are not constant but evolve with the local structural and energetic landscape, as well as with AC field duration and temperature.^51^ As a result, the effective activation energy extracted from the Arrhenius plots varies with these parameters, causing deviation from simple linear behavior. This complex, non-monotonic response is consistent with prior reports on polymer nanocomposites and hopping-dominated conduction, where structural relaxation, interfacial polarization, and charge accumulation produce temperature- and frequency-dependent effects.^63^ The increase in temperature from 25 °C to 40 °C enhances electrical conductivity. This can be explained by the fact that temperature elevation and the influence of the AC electric field stabilize the interfacial structure, thereby facilitating charge carrier hopping.^48^ It is important to note that two different semiconductor materials were used as fillers, which also contribute to the involvement of band conduction mechanisms. Over time, however, conductivity decreases. As the exposure duration to the electric field increases, structural fatigue and the formation of localized traps occur due to the AC field influence, which weakens the hopping process of charge carriers. This leads to a reduction in conductivity.

Next, within the framework of the Correlated Barrier Hopping (CBH) model, we outline the calculation of key parameters such as the maximum potential barrier height (*W*_m_), hopping distance (*R*_*ω*_), and density of localized states (*N*).^64–67^

According to the CBH model, the height of the potential barrier is given by:1
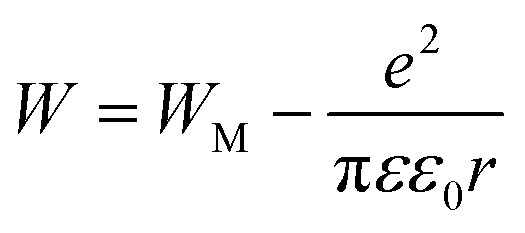
here, *W*_M_ is the maximum height of the potential barrier, *ε* is the dielectric permittivity of the material, *ε*_0_ is the dielectric constant of vacuum, *r* is the distance between two localized states (*i.e.*, the hopping distance), *n* is the number of electrons involved in the hopping process (for polarons *n* = 1 and for bipolarons *n* = 2).

The frequency exponent *s* can be approximately calculated as follows:^64^2
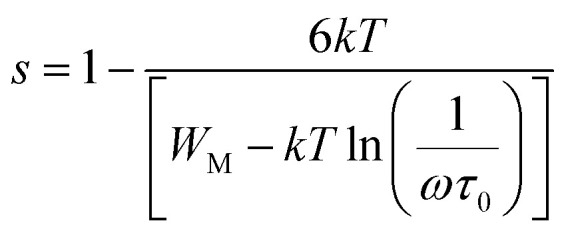


According to the CBH model, the hopping distance *R*_*ω*_ of charge carriers is defined by the following expression:3
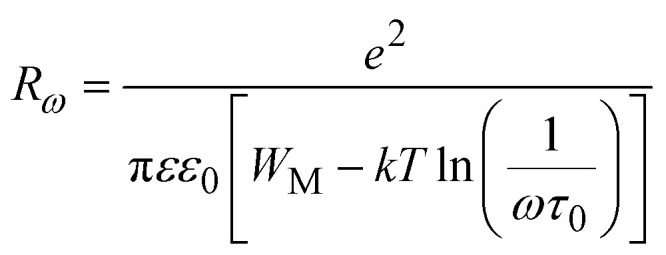
where: *τ*_0_ is the inverse of the atomic vibration frequency (approximately (∼10^−13^ s)), *ω* = 2π*f*_ω_ is the angular frequency of the applied electric field, *ε* is the dielectric permittivity of the material, and *W*_M_ is the maximum potential barrier height. *k*_B_ is the Boltzmann constant, *T* is the absolute temperature in K.

Based on the CBH model, the AC electrical conductivity can be expressed as:^65–68^4
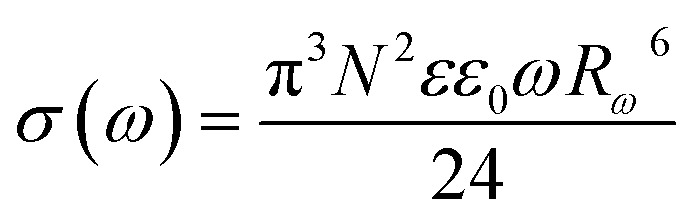
where: *σ*_ac_(*ω*) is the frequency-dependent conductivity, *k* is the Boltzmann constant, *T* is the absolute temperature, *N*(*E*_F_) is the density of localized states at the Fermi level, and *R*_*ω*_ is the hopping distance.
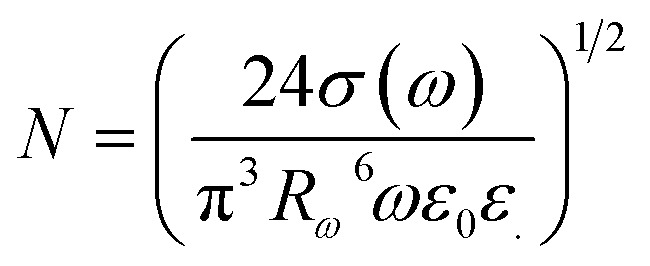


For all CBH calculations, the following constants and conversion factors were used:– *ε*_0_ = 8.854 × 10^−12^ F m^−1^– *e* = 1.602 × 10^−19^ C– *k* = 1.381 × 10^−23^ J K^−1^– *τ*_0_ = 10^−13^ s–1 eV = 1.602 × 10^−19^ J– Frequency (*f*) = 5000 Hz

The dielectric constant *ε* was determined from the measured real permittivity (*ε*′) at the same frequency.

**Fig. 6:**
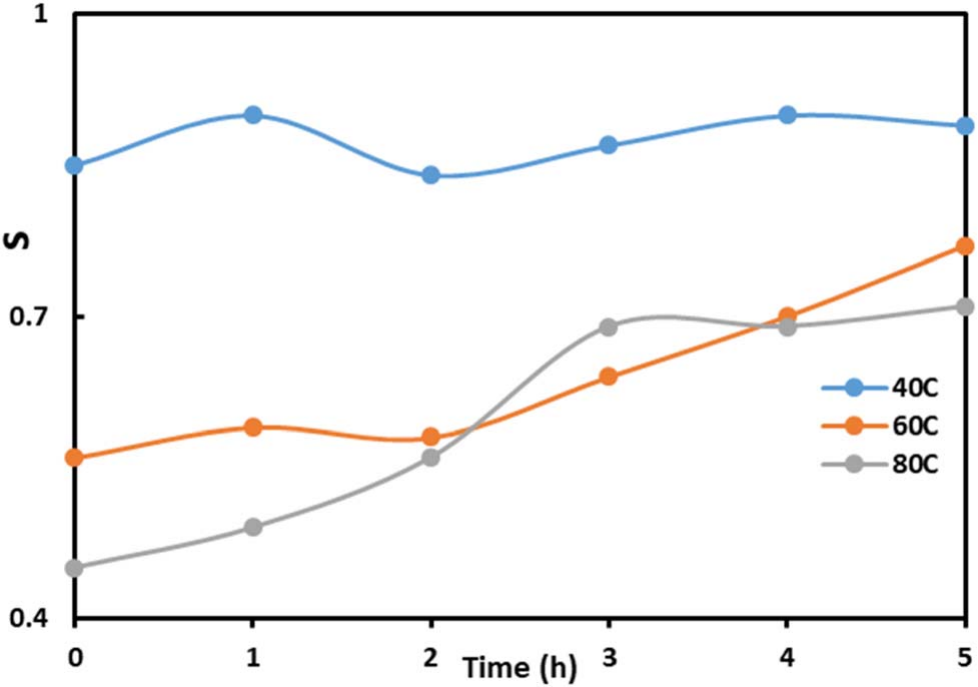
Variation of *s* with AC field exposure duration at different temperatures.

The dependence of *s* on the AC field exposure duration at all three temperatures is shown in Fig. 6.

From the graph, it is evident that *s* increases with time at all three temperatures, although the initial values differ. At higher temperatures, the initial *s* is smaller. This indicates that under short-term AC field exposure at high temperatures, electrons predominantly pass *via* Quantum Mechanical Tunneling (QMT). At high temperatures and short AC-field durations, electrons may not acquire sufficient thermal energy to overcome the energy barrier *via* hopping, as the applied temperature is not sufficiently high. In this case, electrons move between localized states through tunneling, resulting in a lower initial *s*. As the AC-field is applied for longer durations, electrons gain enough thermal energy to perform hopping, leading to an increase in *s*, *i.e.*, the CBH mechanism becomes dominant.

Based on the experimental data (Fig. 4), the system parameters for the PVA + 3wt% GO + 2wt% FeGaInS_4_ composites were calculated 5 × 10^3^ Hz frequency and temperatures of 40 °C, 60 °C, and 80 °C using eqn (1)–(4). The calculated values are summarized in Table 1.

**Table 1:** Temperature-dependent dielectric parameters of PVA + 3wt% GO + 2wt% FeGaInS_4_ composites at 5 × 10^3^ Hz

*T* (°C)	*s*	*τ*_0_ (s)	*ω* (Hz)	*ε*	*σ*_ac_ × 10^−7^ (Ω)	*W*_M_ × 10^−19^ (J)	*R*_*ω*_ (nm)	*N*(m^−3^)
40	0,89 ± 0.03	10^−13^	5000	14.2 ± 0.4	2.5 ± 0.2	2.4 ± 0.1	0.8 ± 0.05	5.5 ± 0.3 × 10^26^
60	0,77 ± 0.02	10^−13^	5000	12.5 ± 0.3	4.1 ± 0.2	1.2 ± 0.1	3 ± 0.2	1.7 ± 0.2 × 10^25^
80	0.71 ± 0.02	10^−13^	5000	17.3 ± 0.5	4.3 ± 0.2	1 ± 0.1	4.4 ± 0.3	3.6 ± 0.2 × 10^24^

The s-parameter shows a decreasing trend with increasing temperature. This behavior is explained by the Correlated Barrier Hopping (CBH) model. It should be noted that in the Quantum Mechanical Tunneling (QMT) model, the *s*-parameter remains almost constant with temperature, whereas in the CBH model, *s* decreases as the temperature rises. This difference is related to the reduction of the barrier energy with temperature in the CBH mechanism, which facilitates the hopping of electrons. As a result, the correlation between charge carriers weakens, allowing more localized states to participate in the conduction process.^67^

This behavior is consistent with trends reported in the literature for PVA-based systems.^24,25^ At low temperatures, the structure of the PVA-GO-FeGaInS_4_ composite is denser and more stable; therefore, the potential barriers are high, meaning that the activation energy required for electron hopping is large. As the temperature increases, segmental mobility in the polymer matrix and structural rearrangements at the GO-FeGaInS_4_ interfaces lead to a reduction in the barrier height (*W*_M_). This facilitates electron hopping to neighboring localized states and consequently increases the a.c. conductivity (*σ*_ac_).

The density of localized states at the Fermi level, *N*(*E*_F_), experimentally shows a decreasing trend with increasing temperature. This decrease is associated with the homogenization of the system, *i.e.*, some local defects and traps are passivated under thermal and AC field effects. As the temperature rises, the energies of some localized states merge, and a portion of them transition into the conduction band, no longer behaving as “active” traps. According to the CBH model, an increase in temperature also leads to an increase in the hopping distance (*R*_*ω*_) and the filling of localized states; as a result, the conductivity increases while the density of active states at the Fermi level *N*(*E*_F_) decreases.^67^

At 40 °C: The temperature is relatively low, so electrons do not acquire sufficient thermal energy during short-term AC field exposure. As the exposure duration increases, electrons gradually gain enough energy to perform hopping, enabling more transitions between localized states. Consequently, *N*(*E*_F_) increases over time, reflecting enhanced utilization of available localized states.^66,67^

At higher temperatures (*e.g.*, 60 °C and 80 °C): Electrons are initially thermally energized, and hopping is already active. With prolonged AC field exposure, some localized states become depleted or fully occupied, resulting in a decrease in the effective *N*(*E*_F_). This indicates that extended AC field application reduces the efficiency of the hopping mechanism at higher temperatures.^66,67^

Moreover, as the temperature approaches the polymer's glass transition temperature (*T*_g_), segmental mobility and carrier kinetic energy further increase, enabling hopping to higher energy levels and longer distances.^67^ Accordingly, *R*_*ω*_ shows a clear temperature-dependent increase, accompanying both enhanced conductivity and a reduced s-parameter. For the studied PVA-GO-FeGaInS_4_ composite under a 5000 Hz AC field (*τ*_0_ = 10^−13^), *s* decreases from 0.89 at 40 °C to 0.71 at 80 °C. Simultaneously, the maximum potential barrier *W*_M_ rises from 2.5 × 10^−19^ J to 4.3 × 10^−19^ J, the hopping distance *R*_*ω*_ increases from 2.4 nm to 4.4 nm, and the density of localized states *N*(*E*_F_) decreases from 5.5 × 10^26^ m^−3^ to 3.6 × 10^24^ m^−3^. The AC conductivity correspondingly increases from 2.5 × 10^−7^ Ω^−1^ to 4.3 × 10^−7^ Ω^−1^.

In general, the combined effects of thermal activation and AC field exposure—interpreted *via* time–temperature superposition—enhance segmental mobility in the polymer chains and promote the stabilization of interfacial regions.^69,70^ The observed trends in the frequency exponent *s* under AC field exposure are well captured by the CBH and NSPT models: *s* initially shows high values due to structural reorganization but gradually stabilizes as the system approaches equilibrium. The increase in *W*_M_ under field application reflects the formation of localized trap states, a behavior also reported for PMMA/SiO_2_ composites.^71^ Furthermore, the evolution of *R*_*ω*_ and *N*(*E*_F_) indicates the development of a more regular and stabilized interfacial structure resulting from prolonged AC field exposure.^72^

Overall, these observations confirm that the temperature-dependent electrical conduction behavior of the PVA-GO-FeGaInS_4_ composite is consistent with the CBH model, while also demonstrating the influence of prolonged AC field exposure on structural stabilization and hopping dynamics.

**Fig. 7:**
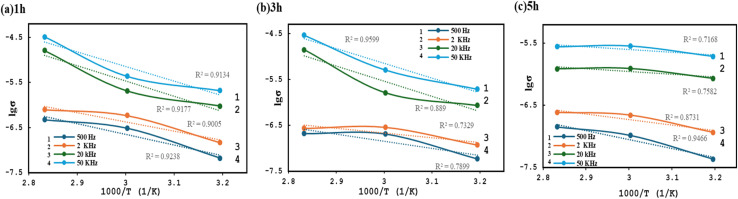
Temperature dependence of the electrical conductivity of PVA/GO/FeGaInS_4_ nanocomposites under different AC field exposure durations: (a) 1 h, (b) 3 h, and (c) 5 h, measured at frequencies of 500 Hz, 2 kHz, 20 kHz, and 50 kHz.

Fig. 7 illustrates the temperature dependence of electrical conductivity for the composite under different AC field exposure durations (1, 2, and 5 hours) and frequencies (500 Hz, 2 kHz, 20 kHz, and 50 kHz).

At 1 hour of AC field exposure, the log *σ vs.* 10^3^/*T* plots at 500 Hz and 2 kHz within the temperature range of 313–333 K are positioned lower on the graph, indicating higher activation energies at these frequencies. This behavior suggests a reduced rate of carrier hopping between localized states, leading to a decline in AC conductivity (Fig. 7a).

In contrast, at higher frequencies of 20 kHz and 50 kHz, the initial conductivity values are significantly higher, indicating that the system exhibits conductive behavior even at lower temperatures. At these frequencies, the temperature dependence of conductivity becomes less pronounced, and the slope of the log *σ* ∼10^3^/*T* plots becomes flatter, suggesting a reduction in activation energy. This phenomenon is consistent with the CBH model, in which charge carriers hop over lower energy barriers at high frequencies.^73^

At 3 hours of exposure (Fig. 7b), the slope remains steep at 500 Hz, indicating that the energy barriers for carrier hopping have not yet been fully suppressed. However, at 2 kHz, the slope flattens, implying a decrease in activation energy and improved interfacial stability.

At 20 kHz and 50 kHz, log *σ* reaches a maximum, indicating that interfacial polarization—particularly the Maxwell–Wagner–Sillars effect—has reached its peak.^65–67^ At this stage, micromolecular polarization is well-developed, and the system approaches a saturation state, wherein prolonged AC field exposure no longer enhances conductivity.

In Fig. 7c, the flattened slopes for 500 Hz and 2 kHz reflect stable hopping processes occurring over lower-energy barriers. According to the CBH model, this can be attributed to the optimization of hopping distances (*R*_*ω*_) and the reduction of energy barriers, resulting in maximum conductivity.

At 20 kHz and 50 kHz, the conductivity appears to be saturated, and further exposure to the AC field results in no significant change; as the temperature increases, the conductivity remains nearly constant and no distinct peak is observed. This behavior indicates a high degree of structural stability.

Based on the experimental data (Fig. 7), the activation energy (*E*_a_) was calculated for the samples exposed to the AC field for 1, 3, and 5 hours, at 500 Hz and 50 kHz, within the temperature range of 313–333 K, and the corresponding values are summarized in Table 2.

**Table 2:** Activation energy (*E*_a_) of the composite as a function of AC field exposure time and frequency

AC field exposure duration (hours)	Frequency	Activation energy (*E*_a_) (eV)	*R* ^2^
1	500Hz	0.75	0.9134
	50 kHz	0.33	0.95
2	500Hz	0.69	0.959
	50 kHz	0.44	0.789
3	500Hz	0.40	0.7168
	50 kHz	0.31	0.946

At 500 Hz, the activation energy (*E*_a_) decreases significantly with increasing AC field exposure duration: from 0.75 eV to 0.69 eV and then to 0.40 eV. This reduction is attributed to the gradual reorganization of the polymer composite structure under prolonged external AC field influence, resulting in more stable and energetically favorable interfacial regions. Under such conditions, the energy barrier for charge carrier hopping between localized states decreases, thereby enhancing the system's conductivity.

At 50 kHz, the activation energy (*E*_a_) exhibits a more complex, biphasic behavior. Initially, *E*_a_ increases from 0.33 eV to 0.44 eV, which can be attributed to restructuring in the interfacial layers and a temporary rise in localized traps induced by the AC field. Additionally, at this higher frequency, multiple hopping mechanisms may simultaneously contribute to the conduction process. Upon further prolongation of the field exposure, *E*_a_ decreases to 0.31 eV, indicating the formation of more stable interfacial structures and the optimization of hopping pathways within the composite.

These changes induced by the AC field are consistent with the Correlated Barrier Hopping (CBH) model. Prolonged application of the field promotes the formation of optimal distances and energy levels for charge transfer at the polymer–filler interfaces, resulting in a reduction of activation energy and enhanced efficiency of the hopping conduction process.

## 4 Conclusion

In this work, PVA-based nanocomposites incorporating layered FeGaInS_4_ and GO fillers were successfully synthesized and systematically investigated to elucidate the effects of temperature and AC field exposure duration on their dielectric behavior.

XRD analysis confirmed that the FeGaInS_4_ crystalline phase was preserved within the composite structure without any detectable phase transformation or lattice distortion, while the absence of characteristic GO peaks indicated its high degree of exfoliation and homogeneous dispersion within the PVA matrix.

Dielectric spectroscopy measurements demonstrated that the dielectric constant (*ε*′) exhibited higher values at low frequencies due to dominant Maxwell–Wagner–Sillars interfacial polarization and decreased with increasing frequency as dipolar polarization became prevalent. The dielectric constant increased with rising temperature, which was attributed to enhanced molecular mobility of PVA chains, interfacial polarization, and expansion of the interphase regions.

At 40 °C, short-term exposure (up to 2 h) to the AC field improved both the dielectric constant and loss tangent (tg *δ*) as a result of interfacial reorganization and defect relaxation. However, prolonged exposure induced structural fatigue and trap formation, leading to relaxation suppression and a subsequent reduction in dielectric performance. At higher temperatures, such effects became more pronounced, indicating accelerated dielectric aging under combined electrical and thermal stress.

The AC conductivity analysis, interpreted using the CBH model, revealed that thermal activation and electric field exposure reduced the energy barrier for charge carrier hopping and optimized the hopping distance (*R*_*ω*_), resulting in increased conductivity. The temperature-dependent decrease in the frequency exponent (*s*) further confirmed that the conduction mechanism in the studied composites follows the CBH model rather than the QMT model.

Overall, the synergistic incorporation of FeGaInS_4_ and GO into the PVA matrix significantly enhanced interfacial polarization and charge transport dynamics, enabling tunable dielectric properties through thermal and electrical field modulation. These findings suggest that PVA/FeGaInS_4_/GO nanocomposites possess high interphase stability and dielectric adaptability, making them promising candidates for flexible electronic devices, dielectric sensors, and high-performance energy storage applications.

## Author contributions

Zeynab Addayeva – investigation, data curation, writing – review & editing, Mustafa Muradov – conceptualization, methodology, validation, supervision, project administration. Goncha Eyvazova – software, visualization. Namiq Niftiyev – software, writing – original draft, formal analysis, investigation. Faiq Mammadov – resources.

## Conflicts of interest

The authors declare no competing interests.

## Data Availability

All data generated or analyzed during this study are included in this published article.
